# Resistance Mechanisms to Anti-angiogenic Therapies in Cancer

**DOI:** 10.3389/fonc.2020.00221

**Published:** 2020-02-27

**Authors:** Yolla Haibe, Malek Kreidieh, Hiba El Hajj, Ibrahim Khalifeh, Deborah Mukherji, Sally Temraz, Ali Shamseddine

**Affiliations:** ^1^Division of Hematology/Oncology, Department of Internal Medicine, American University of Beirut-Medical Center, Beirut, Lebanon; ^2^Department of Experimental Pathology, Immunology and Microbiology, American University of Beirut-Medical Center, Beirut, Lebanon; ^3^Department of Pathology and Laboratory Medicine, American University of Beirut Medical Center, Beirut, Lebanon

**Keywords:** VEGF, VEGF-R, bevacizumab, colorectal cancer, angiogenesis, resistance mechanisms

## Abstract

Tumor growth and metastasis rely on tumor vascular network for the adequate supply of oxygen and nutrients. Tumor angiogenesis relies on a highly complex program of growth factor signaling, endothelial cell (EC) proliferation, extracellular matrix (ECM) remodeling, and stromal cell interactions. Numerous pro-angiogenic drivers have been identified, the most important of which is the vascular endothelial growth factor (VEGF). The importance of pro-angiogenic inducers in tumor growth, invasion and extravasation make them an excellent therapeutic target in several types of cancers. Hence, the number of anti-angiogenic agents developed for cancer treatment has risen over the past decade, with at least eighty drugs being investigated in preclinical studies and phase I-III clinical trials. To date, the most common approaches to the inhibition of the VEGF axis include the blockade of VEGF receptors (VEGFRs) or ligands by neutralizing antibodies, as well as the inhibition of receptor tyrosine kinase (RTK) enzymes. Despite promising preclinical results, anti-angiogenic monotherapies led only to mild clinical benefits. The minimal benefits could be secondary to primary or acquired resistance, through the activation of alternative mechanisms that sustain tumor vascularization and growth. Mechanisms of resistance are categorized into VEGF-dependent alterations, non-VEGF pathways and stromal cell interactions. Thus, complementary approaches such as the combination of these inhibitors with agents targeting alternative mechanisms of blood vessel formation are urgently needed. This review provides an updated overview on the pathophysiology of angiogenesis during tumor growth. It also sheds light on the different pro-angiogenic and anti-angiogenic agents that have been developed to date. Finally, it highlights the preclinical evidence for mechanisms of angiogenic resistance and suggests novel therapeutic approaches that might be exploited with the ultimate aim of overcoming resistance and improving clinical outcomes for patients with cancer.

## Introduction

### Angiogenesis

Angiogenesis is the process of formation of new blood vessels from pre-existing vessels. It is a highly regulated process that involves migration, growth, and differentiation of endothelial cells (ECs). This regulated mechanism is crucial in embryonic development, wound healing, and reproduction (1). Nonetheless, alterations in any of its regulatory pathways may lead to metabolic diseases, cardiovascular disorders, diabetic retinopathy, psoriasis, systemic lupus erythematosus, and importantly tumor growth and metastasis (2–5).

**Figure 1:**
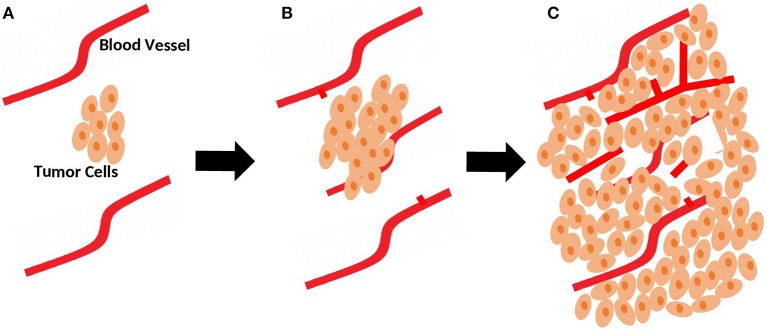
Role of sprouting angiogenesis in tumor growth. **(A)** During early stages of development, tumor is still small in size and relies on local existing blood vessels for oxygen and nutrients supply. **(B)** As the tumor grows, sprouting of new vessels from local existing blood vessels occurs to fulfill the need for more oxygen and nutrients supplies. **(C)** Sprouting angiogenesis results in a more complex network of vasculature to provide adequate blood supply for the growing tumor.

In the avascular phase, tumor growth is usually restricted in size due to a balance between pro-angiogenic and anti-angiogenic factors that control vascular homeostasis (6). Beyond a few millimeters in size, solid tumors build, and increase their own blood supply to provide adequate oxygen and nutrients (Figure 1). This process, referred to as the angiogenic switch, from an avascular state to an angiogenic phase, is crucial for tumors to grow and continue unrestricted proliferation (7). Hence, unlike normal physiological processes favoring negative regulation of angiogenesis, tumors favor its upregulation.

Multiple non-mutually exclusive mechanisms have been described as major players in tumor neovascularization. These include sprouting angiogenesis, non-sprouting angiogenesis, vasculogenesis, vasculogenic mimicry, and intussusception. Sprouting angiogenesis, however, remains the most well-studied mechanism used by tumor cells to produce their vasculature (8). Due to the importance of this latter process in tumor cell growth, invasion, and extravasation, different angiogenesis inhibitors (AIs) have been developed.

In this review, we will discuss the different driver molecules promoting angiogenesis in cancer. These include the angiogenic or angiostatic chemokines, the contribution of the endothelial progenitor cells (EPCs), the tumor vasculogenic mimicry, the markers for tumor-derived ECs, and pericytes. We will also provide an overview on the clinically tested anti-angiogenic drugs slowing down angiogenesis and leading to tumor starvation. Finally, the resistance mechanisms arising in cancer cells against these drugs and the potential therapeutic solutions will be discussed.

### Angiogenesis: Pathophysiology During Tumor Growth

Unlike normal angiogenesis and neovascularization, tumor angiogenesis is an uncontrolled and disorganized process. It results in vessels with thin walls, incomplete basement membranes, and atypical pericytes (8). Since the needs of rapid tumor cell proliferation surpass the capacity of host vasculature, hypoxia and low supplies of nutrients characterize early stages of tumor development. Hypoxia triggers the expression of pro-angiogenic factors such as vascular endothelial growth factor (VEGF) and platelet-derived growth factor (PDGF) (9–11).

**Figure 2:**
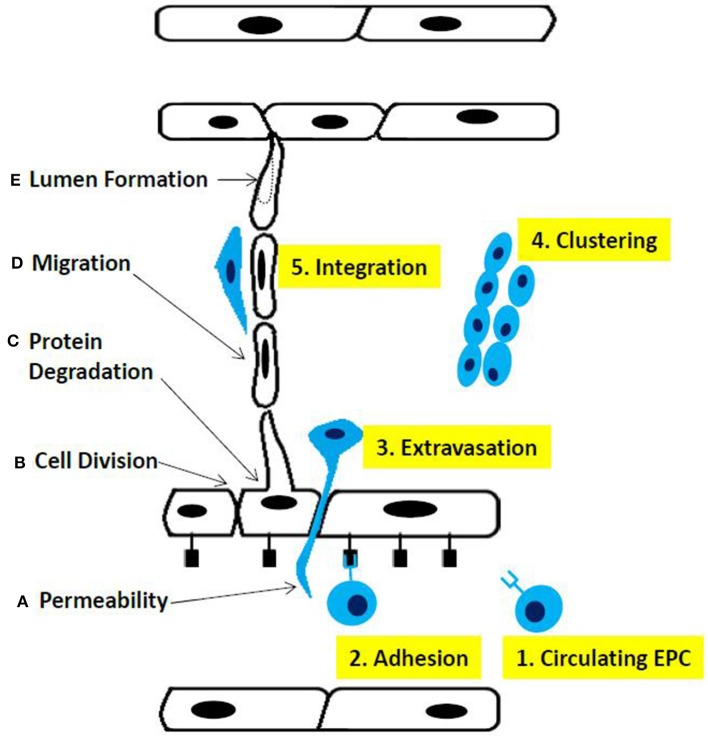
Phases of sprouting angiogenesis. **(A)** increased permeability across the endothelial cell layer, **(B)** cell division, **(C)** proteolysis of basement membrane components, **(D)** migration ofthe endothelial cells, and **(E)** lumen fonnation. Altematively, (1) circulating endothelial progenitor cells contribute to the sprouting mechanism, (2) adhere to endothelial cells, (3) extravagate through the endothelial cell layer, (4) cluster together, and (5) integrate into the sprout fonned by endothelial cells.

Matrix metalloproteinases (MMPs) secreted by tumor cells degrade the basement membrane as a first essential step to initiate angiogenesis (12). This alters cell-cell interactions and facilitates the migration of ECs through the created gap into the tumor mass, which in turn results in the proliferation and formation of new blood vessels, followed by vessel pruning and pericyte stabilization (Figure 2).

### Angiogenesis: Regulation

Angiogenesis is a tightly balanced mechanism regulated by both pro-angiogenic and anti-angiogenic factors (13). In malignant tumors, this balance is shifted toward a pro-angiogenic milieu to maintain sustainable angiogenic processes (14). Involved soluble growth factors include VEGF, PDGF, fibroblast growth factor (FGF)-2, angiopoietins (Angs), transforming growth factors (TGFs)- beta and alpha, and epidermal growth factors (EGF). Insoluble membrane-bound factors include integrins, ephrins, cadherins, MMPs, and hypoxia inducible factor-1 (HIF-1).

From these, VEGF was broadly studied and shown to significantly contribute to the induction and progression of angiogenesis (15). We will start by listing the different members of the VEGF family. In the following sections, a general overview on the role of the other angiogenic factors in normal and tumor angiogenesis will be described. In addition, direct and indirect angiogenesis inhibitory mechanisms will be discussed.

### Vascular Endothelial Growth Factor Family

**Figure 3:**
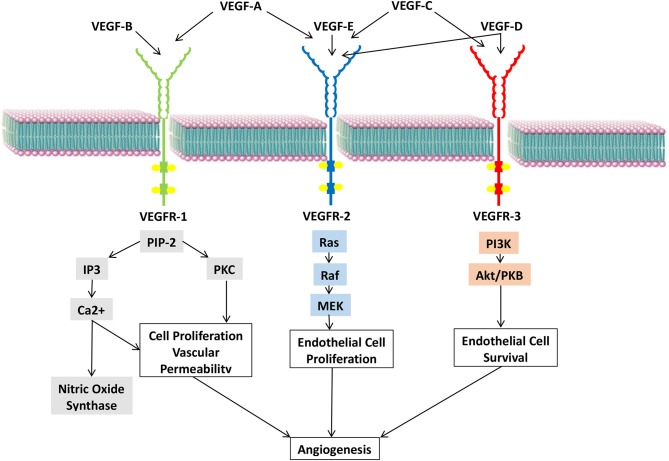
VEGF ligands, their receptors, and respective signaling pathways.

The VEGF family comprises seven members, VEGFs A to F and placenta growth factor (PGF) (16). These members are ligands that interact with multiple receptors present on the vascular endothelium (17) (Figure 3).

#### Vascular Endothelial Growth Factor A

VEGF-A is the most potent angiogenic factor that is encoded by a gene located on the short arm of chromosome six (18). Its interaction with the transmembrane tyrosine kinase receptors, VEGF receptors (VEGFRs)-1 and 2, and their co-receptors, NRPs-1 and 2, present on vascular ECs results in the dimerization and phosphorylation of intracellular receptors (19). This further activates downstream signaling cascades involving phosphatidylinositol 3-kinase (PI3K)/protein kinase B (Akt), mitogen-activated protein kinase (MAPK), and extracellular regulated kinase (ERK) (20, 21).

VEGF-A expression is stimulated by hypoxia, growth factors, and cytokines such as IL-1, EGFs, PDGFs, and tumor necrosis factor (TNF)-α (16). It was noted in most solid tumors and some hematologic malignancies (20). VEGF-A is considered the backbone of angiogenesis during physiologic as well as pathologic processes. The deletion of one or both VEGF-A alleles in mouse pre-clinical models resulted in either vascular abnormalities or complete absence of vasculature leading to death (22). Interestingly, a striking positive correlation between the level of VEGF-A expression, tumor progression, and cancer patients' survival was observed (23, 24).

#### Vascular Endothelial Growth Factor B

VEGF-B is encoded by a gene located on chromosome eleven. It differs from VEGF-A by its promotor region (25, 26). It was found to be upregulated in many types of tumors including prostate, kidney, and colorectal cancers (CRCs) (27, 28). Since the VEGF-B promoter lacks the HIF-1 and AP-1 sites found in the VEGF-A promotor, stimuli such as hypoxia or cold do not induce VEGF-B expression (29, 30).

A study was conducted to explore the role of VEGF-B in cancer development. Results revealed that VEGF-B-deficient transgenic mice with pancreatic endocrine adenocarcinoma had larger tumors compared to transgenic expression of VEGF-B but no difference in tumor vasculature (31). In addition, knockout studies have highlighted the role of VEGF-B in inflammatory angiogenesis and regeneration of coronary collaterals through arteriogenesis (32, 33).

#### Vascular Endothelial Growth Factors C, D, and E

The VEGF-C encoding gene is located on chromosome four (34–36). Experiments performed on transgenic mice demonstrated the ability of VEGF-C to induce selective lymphangiogenesis without accompanying angiogenesis (37). Several studies showed a positive correlation between VEGF-C expression, lymphatic invasion, metastasis, and survival in cancer patients. For instance, while the 2-year survival rate of patients with uterine cervical cancers with high VEGF-C level in metastatic lymph nodes was 38%, that of patients with normal levels was 81% (38, 39).

VEGF-D is closely related to VEGF-C with which it shares 61% homology (40). Similar to VEGF-C, VEGF-D can bind and activate the VEGFRs 2 and 3 (41, 42). Depending on the activated receptor, separate downstream cascades are activated to induce the growth and proliferation of ECs in the vascular and lymphatic systems (43). As such, VEGF-D activity is crucial for hypoxia-induced vascular development (44) in melanoma, lung, breast, pancreatic, and esophageal cancer (43, 45–48).

VEGF-E is a potent angiogenic factor. Its isoform, VEGF-E nz-7, binds with high affinity to VEGFR-2 to stimulate efficient angiogenesis and increase vascular permeability (49).

#### Placental Growth Factor

PlGF is a member of the VEGF subfamily that binds to VEGFR-1 and its co-receptors, NRP-1 and 2. PlGF/VEGFR-1 signaling activates the downstream PI3K/Akt and p38 MAPK pathways independent of VEGFA signaling (50, 51). This stimulates the growth and migration of ECs, macrophages, and tumor cells (52, 53).

Upregulation of PlGF expression has been observed in tumors resistant to anti-VEGF therapy suggesting that PlGF might serve as a promising therapeutic target in this setting (54–57). In addition, PlGF knockout (pgf^−/−^) mice were noted to have normal embryonic angiogenesis and impaired pathological angiogenesis following exposure of their tumors to ischemia (58). This suggests that by neutralizing PlGF, pathological angiogenesis can be inhibited without affecting normal blood vessels (59).

## Currently Approved Anti-angiogenic Therapies

Since sprouting angiogenesis plays an essential role in tumor growth, invasion, progression, and metastasis, targeting this process may potentially halt the growth and spread of cancer (60). Table 1 lists antiangiogenic agents approved for clinical use and their targets.

**Table 1:** List of some FDA-approved anti-angiogenic agents.

**Drug name**	**Drug class**	**Targets**	**Indications**
Bevacizumab	VEGF-A antibody	VEGF-A	Metastatic CRC
			Metastatic RCC
			Metastatic Ovarian cancer
			recurrent glioblastoma
Ramucirumab	VEGFR2 antibody	VEGFR2	Metastatic Gastric or GEJ
			Metastatic CRC
Aflibercept	VEGF-Trap	VEGF-A VEGF-B	Metastatic CRC
Sunitinib	Tyrosine Kinase Inhibitor	All VEGFRs	PNET
		FGFR1, cKIT, PDGFR	Metastatic GIST
			Metastatic RCC
Sorafenib	Tyrosine Kinase Inhibitor	ALL VEGFRs	Metastatic RCC
		FGFRs, PDGFRs	Metastatic HCC
		FLT3	Metastatic thyroid carcinoma
Pazopanib	Tyrosine Kinase Inhibitor	All VEGFRs	Metastatic RCC
		FGFR2, cKIT	Metastatic soft tissue sarcoma
		PDGFR,FLT3	
Axitinib	Tyrosine Kinase Inhibitor	All VEGFRs	Metastatic RCC
		PDGFRs, cKIT	
Cabozantinib	Tyrosine Kinase Inhibitor	All VEGFRs,	Metastatic medullary thyroid carcinoma
		cKIT, cMET, Ret	Metastatic HCC
Lenvatinib	Tyrosine Kinase Inhibitor	All VEGFRs,	Metastatic thyroid cancer
		PDGFRs,FGFR1	Metastatic HCC

Angiogenesis inhibitors (AIs) are classified into direct and indirect agents. Direct endogenous inhibitors target vascular ECs and include endostatin, arrestin, and tumstatin. Unfortunately, phase II or III clinical trials did not result in significant effects on patients (14, 61). In the last decade, a number of molecules have been described, including semaphorins, netrins, slits, and others (62–64). Netrin-1, Netrin-4, and their receptors can have a repulsive or attractive signals in angiogenesis, partially via the regulation of VEGF signaling. There are still some contradictions reported on the positive and negative role of Netrin-1 in regulation of angiogenesis, and studies are still on going to identify its exact role in angiogenesis. Semaphorin-3A and Semaphorin-3E have negative effects on angiogenesis in central nervous system (CNS) and non-CNS tissues.

Indirect AIs target tumor cells or tumor associated stromal cells and include several types (14) (Table 2). They prevent the expression of pro-angiogenic factors or block their activity.

**Table 2:** List of indirect angiogenesis inhibitors.

**Type**	**Drug name(s)**
VEGF-targeted therapy	Bevacizumab
	Sunitib
	Sorafenib
FGF-targeted therapy	Ponatinib
	Pintedanib
	Dovitinib
Oncogene-targeted therapy	Dasatinib
	Tipifarnib
	Bortezomib
Matrix degrading and remodeling-targeted therapy	DX-2400
	PI-88
Tumor-associated stromal cell-targeted therapy	Zoledronic acid
Cell adhesion molecules-targeted therapy	Cilengitide
	Zolociximab
Inflammatory angiogenesis-targeted therapy	Ibuprofen
	Repertaxin
	Celecoxib
Conventional chemotherapeutic agents	Cyclophosphamide

Among the AIs, VEGF inhibitors were extensively studied and reached phase III clinical trials. They caused a modest increase in overall survival (OS) (65). Bevacizumab (BVZ), a humanized anti-VEGF monoclonal antibody, was the first drug to be approved by the Food and Drug Administration (FDA) for the treatment of metastatic colon, ovarian, renal, non-squamous cell lung cancer (NSCLC), and glioblastoma mutliforme (GBM) (66, 67). It failed to show clinical significance when used as monotherapy, except in GBM. In contrast, its clinical benefits were evident in association with other chemotherapeutic agents. For instance, since the tumor vasculature induced by VEGF is usually tortuous and dysfunctional, the use of BVZ was thought to normalize the blood vessel texture. It was also hypothesized that the combination of BVZ and chemotherapy increases the delivery of the chemotherapeutic agent to the cancer tissue by increasing its blood flow (68, 69). However, contrary evidence was reported by a decrease in cytotoxic drug delivery to tumors following treatment with AIs (70). Such inconsistency could be due to differences in blood vessel setups among various cancer types (71, 72). BVZ combined with chemotherapy was also studied in the adjuvant setting in colorectal cancer (CRC), but it failed to prove any clinical significance compared to chemotherapy alone in two phase III clinical trials (73–75).

Aflibercept is a soluble VEGF decoy receptor that consists of the extracellular domains of VEGFRs 1 and 2 and the Fc portion of human IgG1. It was FDA approved for the treatment of metastatic CRC in combination with 5-fluorouracil, leucovorin, and irinotecan in 2012 (76). Owing to its structure, Aflibercept can neutralize both, VEGF and PlGF (77). Compared to treatment with BVZ, the use of Aflibercept in patient-derived xenograft models resulted in higher tumor suppressive activity (78). Unfortunately, neutralizing both, PlGF and VEGF, had a minimal effect on tumor suppression *in vivo* (79). In a phase I clinical trial, relapsing GBM patients treated with BVZ monotherapy were compared to those treated with the combination of an anti-PlGF agent and BVZ. Similar results were obtained with no added benefit in the combination arm (80).

Unlike BVZ and Aflibercept, tyrosine kinase inhibitors, which are small molecules able to interact with the kinase domain on the VEGFRs, showed a remarkable clinical benefit when used as single agents, and with no added value when combined with chemotherapy. This was reported in the treatment of renal cell carcinoma (RCC), hepatocellular carcinoma (HCC), thyroid cancer, gastrointestinal stromal tumor (GIST), and pancreatic neuroendocrine tumor (PNET) (81).

## Mechanisms of Resistance to Anti-Angiogenic Therapies and Ways to Overcome them

**Figure 4:**
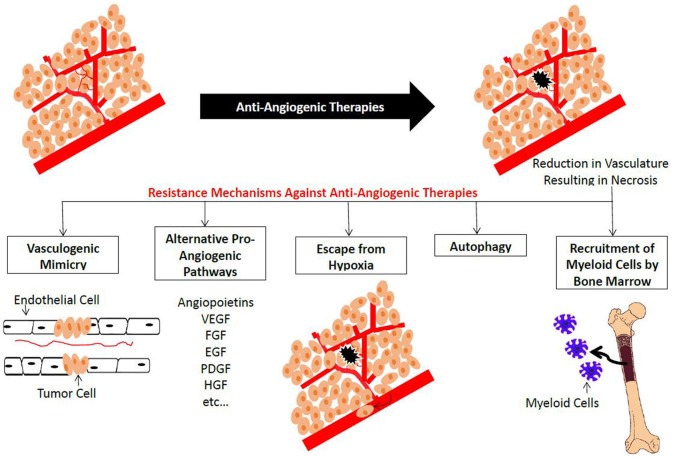
Summary of plausible resistance mechanisms to Anti-angiogenic Agents. Treatment with anti-angiogenic agents results in a reduction in the blood vessel network. This new hypoxic condition results in the activation of vascular mimicry, altemative pro-angiogenic pathways, recruitment of bone man·ow-derived EC precursors and myeloid cells, as well as cell survival mechanisms such as autophagy.

Although anti-angiogenesis therapies may prolong progression-free survival (PFS), they have limited impact on overall survival (OS) and do not constitute a permanent cure in RCC, CRC, or breast cancer (73, 75, 82, 83). This limited clinical significance might be due to different innate and acquired molecular resistance mechanisms with no clear genetic explanations (65). Hypoxia plays an important role in tumor resistance to chemotherapeutic agents favoring more aggressive metastatic disease and hence worse prognosis. HIF-1 plays a critical role in resistance to anti-angiogenic therapy and is the main survival factor used by cancer cells to adapt to oxygen deprivation (84, 85). In this section, an overview on different mechanisms of resistance to anti-angiogenic therapies in the clinical and preclinical settings will be discussed (Figure 4) and the ways to overcome them will be provided (Table 3). Some of these mechanisms are likely influenced by hypoxia. These include the production of alternative proangiogenic factors, the recruitment of BM-derived cells, the vasculogenic mimicry, as well as the increased tumor cell invasiveness and metastatic behavior.

**Table 3:** List of mechanisms of resistance to anti-angiogenic therapies and ways to target them along with the outcomes associated with each approach.

**Mechanisms of resistance to anti-angiogenic therapies**		**Targeting resistance mechanisms**	**Outcome(s) Reference(s)**	
**I**.	**Increased tumor invasiveness and metastasis**			
		• Crizotinib: a dual c-Met and ALK inhibitor	• Reversal of sunitinib-induced invasion	(86–88)
			• Reversal of expression of EMT markers in different models	
		• Adenoviral Sema3A expression	• Impressive increase in median survival and a reduction in metastasis and hypoxia	(89)
			• Normalization of tumor vasculature	
		• Gemcitabine and Topotecan	• Reversal of sunitnib-induced metastasis and a reduction primary tumor growth	(90)
			• Topotecan: inhibition of HIF-1a accumulation –> preventing hypoxia-driven invasiveness	(91)
**II**.	**Redundancy in angiogenic signaling pathways**			
	1. *Angiopoietin*	• VEGF and Ang2 Blockade	• Preclinical studies: suppression of revascularization and tumor progression of cancers resistant to anti-VEGF therapy	(92–95)
	2. *Bombina variegate peptide 8 (Bv8)*	• PKRA7 (Bv8 antagonist)	• Suppression of tumor formation *in vivo* by inhibiting angiogenesis in GBM and infiltration of MDSCs in pancreatic cancer	(96)
	3. *Fibroblast growth factor (FGF)*	• PD173074 (FGFR inhibitor) + BVZ	• Xenografted mouse models with HNSCC: complete regression of tumor	(97)
		• FGF-trap (soluble FGF receptor) + VEGFR2 inhibitor	• Late stage pancreatic islet tumors: complete regression of tumor	(98)
		• Dovitinib or Nintedanib	• Clinical setting: no benefit in patients with recurrence following anti-VEGF therapy	(99, 100)
	4. *Platelet-derived growth factor*	• Sunitinib (VEGFR + PDGFR)	• FDA approval in 2006 for the treatment of metastatic RCC	(101)
		• BVZ + Imatinib (anti-PDGF agent)	• Toxic and not effective against RCC	(102–104)
	5. *Transforming growth factor-β*	• Galunisertib (TGFβRI Inhibitor) + Sorafenib + Ramucirumab	• Currently under evaluation in HCC	
		• PF-03446962 (Anti-TGFβ monoclonal antibody) + Regorafenib	• Currently under evaluation in CRC	
	6. *Matrix metalloproteinases*	• MMP inhibitors	• Phase I clinical trial: Some clinical efficacy in patients with advanced and refractory solid tumors	(105)
**III**.	**Recruitment of bone marrow-derived cells**			
	1.*Myeloid cells*	• SDF1 neutralizing antibody	• Transgenic mouse model of breast cancer: inhibition of MDSC infiltration and angiogenesis	(106).
		• Gemcitabine + Anti-Bv8 monoclonal antibody	• Mice with adenocarcinoma: inhibition of tumor regrowth, angiogenesis, and metastasis	(107)
		• Carlumab (Anti-CCL2 monoclonal antibody)	• Phase I clinical trial: patients with solid tumors with a temporary antitumor activity	(108)
		• Combined ANG2 and VEGFR2 blockade	• Decreased infiltration of TIE2 expressing monocytes and suppression of revascularization and tumor progression	(92)
**IV**.	**Recruitment of local stromal cells**			
	1. *Pericytes*	• Imatinib + SU11248 + Cyclophosphamide + or - an anti-VEGFR agent	• Preclinical study on transgenic mice with cancer: significant improvement in anti-tumor responses	(109)
	1. *Cancer-associated fibroblasts*	• GAL-F2 (Anti-FGF2 monoclonal antibody)	• Neuroblastoma mouse xenograft models: sustained anti-angiogenic effects	(110)
		• Brivanib (Dual VEGFR/FGFR inhibitor)	• Patients with recurrent and persistent endometrial cancer: extension of their progression-free survival	(111)
**V**.	**V Adoption of different neovascularization modalities**			
	1. *Vasculogenic mimicry*	• Anti-CD44 agent	• Ongoing clinical study: Pending	NCT01358903
**VI**.	**Hypoxia caused by anti-angiogenic therapies**			
	1. *Hepatocyte growth factor/tyrosine protein kinase met pathway*	• Onartuzumab (c-MET inhibitor) + BVZ	• Patients with advanced NSCLC: No clinical benefit	(112)
	2. *β1 integrin expression*	• β1 integrin blockade	• Preclinical studies: benefit in BVZ-resistant and non-resistant GBM tumors in xenograft models	(113, 114)

### Hypoxia Caused by Anti-angiogenic Therapies

Treatment with anti-angiogenic agents results in vascular regression and intra-tumoral hypoxia. Several studies have made use of pimonidazole injections, to demonstrate an increase in hypoxic regions in primary tumors following anti-angiogenic treatment (86, 89, 115). Further analysis showed a concomitant increase in HIF-1a expression during treatment.

HIF-1a and hypoxia are known drivers of EMT, a process that promotes tumor metastasis. Upregulation of EMT-related genes, such as Twist and Snail, have been noted following anti-angiogenic treatment. This is in addition to the loss of the epithelial marker, E-cadherin, and the induction of the mesenchymal marker, vimentin (86, 116). Hypoxic environments also induce upregulation of VEGF expression through the upstream transcription factor HIF-1a (117). These factors cause tumors to acquire more angiogenic and invasive capacities, thus promoting metastasis (118).

#### Effect of Hypoxia on the Hepatocyte Growth Factor/Tyrosine Protein Kinase Met Pathway

The increase in tumor invasiveness and metastasis in response to AI-induced hypoxia from anti-angiogenic therapies can be explained by the over-expression of the tyrosine protein kinase, c-MET. For instance, *in vitro* studies revealed a direct positive effect of hypoxia on c-MET and phospho-c-Met expression (87). Other studies confirmed that this promotion of c-MET transcription that follows hypoxic conditions occurs via the direct regulation of HIF-1 (119).

The HGF/c-MET pathway is one of the most investigated signaling pathways in tumors resistant to anti-VEGF therapy. Binding of HGF to c-MET activates MAPK/ERK cascades, STAT3 pathway, PI3K/Akt axis, and/or NF-κB inhibitor-α kinase (IKK)-NF-κB complex (119–121). This usually promotes tumor growth and invasiveness.

VEGF exerts a negative feedback on c-MET activation in a GBM mouse model, resulting in the direct suppression of tumor invasion (122). For instance, compared to GBM patients who were not treated with BVZ, those treated with BVZ had more recurrence rates and their tumors had an upregulation in c-MET expression (123). This increased invasiveness of GBM after BVZ treatment was recently linked to inhibitory actions of VEGF and to the increase in c-Met and phospho-c-Met expression upon treatment (122).

MET activation in response to hypoxia can occur in endothelial cells, as well as in tumor cells or other cells of the tumor microenvironment. In fact, in one study (124) this had very diverse functional impacts.

#### Blocking c-MET to Overcome Resistance to Anti-vascular Endothelial Growth Factor Treatment

To overcome the c-MET protein overexpression that occurs with the neutralization of VEGF by BVZ, the addition of a c-MET inhibitor would be helpful. In the phase III METEOR trial, the administration of the inhibitor of tyrosine kinases including MET, Cabozantinib, after previous vascular endothelial growth factor receptor-targeted therapy in patients with advanced RCC resulted in improved survival (125).

#### Effect of Hypoxia on β1 Integrin Expression

It is thought that the hypoxic microenvironment generated during anti-angiogenic therapy induces HIF-1α expression, thus stimulating β1 integrin expression. β1 integrin is the member that is mostly implicated in cancer treatment resistance, especially that its expression has been upregulated in clinical specimens of BVZ-resistant GBM tumors (126–128). The expression levels of integrins are correlated with disease progression and poor survival of patients (129, 130). Upon interacting with c-MET, integrins ultimately enhance tumor cell invasiveness (113, 131, 132).

#### Blocking β1 Integrin to Overcome Resistance to Anti-vascular Endothelial Growth Factor Treatment

Several preclinical studies have demonstrated benefit from β1 integrin blockade in BVZ-resistant and non-resistant GBM tumors in xenograft models (113, 114).

### Increased Tumor Invasiveness and Metastasis

Despite their overall inhibition of tumor growth, therapeutic AIs were associated with increased local invasiveness and distant metastasis. These phenomena seem to be major contributors to resistance against anti-angiogenesis therapies. They were first described by Ebos et al. and Paez-Ribes et al. in different preclinical models (115, 133).

Angiogenesis blockade enhances tumor invasiveness. For instance, RCC cells demonstrated an accelerated growth capacity and an invasive profile following treatment with BVZ (134). Similarly, GBM cells in mouse models developed enhanced invasiveness following VEGF inhibition (115).

Treatment with AIs also promotes tumor metastatic potential. Treatment with sunitinib has been shown to result in vascular changes that include decreased adherens junction protein expression, reduced basement membrane and pericyte coverage, and increased leakiness (89, 91, 135, 136). These phenotypic changes were observed in both, tumor vessels and normal organ vessels, so they tend to facilitate local intravasation and extravasation of tumor cells, resulting in metastatic colonization (136).

#### Factors Promoting or Affecting Tumor Invasiveness and Metastasis

Increased metastasis and enhanced invasiveness in response to anti-angiogenesis therapy are variable and depend on the treatment type, dose, and schedule. Singh et al. observed that sunitinib and anti-VEGF antibody monotherapy had different effects on mouse tumor models. While treatment with sunitinib enhanced the aggressiveness of tumor cells, using an anti-VEGF antibody did not (91). This was supported by Chung et al. who compared the efficacy of different RTK inhibitors and antibody therapies in murine models (135). While pretreatment with imatinib, sunitinib, or sorafenib enhanced lung metastasis following the injection of 66c14 cells, using an anti-VEGFR2 antibody inhibited the formation of lung nodules (135). Altogether, these results prove that the increased metastasis and enhanced invasiveness that result from use of AIs are largely dependent on treatment type.

Dosing and scheduling of administration of AIs can also induce resistance. Indeed, treatment with short-term and high-dosage sunitinib (120 mg/kg per day) before and after intravenous breast tumor cell inoculation into severe combined immune-deficient mice had the most deleterious effects (133). The high-dose of sunitinib increased tumor growth and enhanced metastasis to the liver and lung, resulting in reduced survival. Although similar results were observed using sorafenib, contradictory results were reported with sunitinib in different studies (115, 133). In fact, treatment with high-dose sunitinib before intravenous inoculation of tumor cells increased metastatic potential of lung cancer cells but not of RCC cells. In contrast, treatment with low-dosage sunitinib (30 and 60 mg/kg per day) did not stimulate metastasis (136).

It was documented that hypoxia and EMT also contribute to the increased invasiveness and metastasis of tumors, and c-Met, Twist, and HIF-1a are the key molecular players (11, 116). In contrast, semaphorin 3A (Sema3A), an endogenous anti-angiogenic molecule, is frequently lost in tumors, resulting in increased invasiveness and metastasis (137).

#### Overcoming Resistance by Targeting Increased Tumor Invasiveness and Metastasis

Different inhibitors of c-Met were tested in preclinical studies and demonstrated promising effects. Crizotinib, a dual c-Met and ALK inhibitor, was effective in reverting sunitinib-induced invasion and metastasis in different models (86–88). Interestingly, this resulted in a reduction in the expression of EMT markers such as Vimentin, Snail, and N-cadherin downstream of c-Met (86, 87). By blocking c-Met and silencing Twist, the master regulator of EMT (138), metastasis was almost fully abrogated in both wild-type and pericyte-depleted tumors (86).

Sunitinib-treated transgenic mice tumors that were subjected to adenoviral Sema3A expression witnessed an impressive increase of 10 weeks in median survival and a reduction in metastasis and hypoxia (89). Normalization of the tumor vasculature was evident, and the expression of EMT markers, including c-Met, were reduced.

Rovida et al. investigated the use of conventional chemotherapeutics to counteract sunitinib-induced metastasis. Gemcitabine and topotecan, but not paclitaxel, cisplatin, and doxorubicin, were effective in reverting sunitinib-induced metastasis and in reducing primary tumor growth (90). Mechanistically, topotecan was shown to inhibit HIF-1a accumulation, thereby preventing hypoxia-driven invasiveness. Gemcitabine was moderately effective in combination with anti-VEGF antibody therapy in an established pancreatic ductal adenocarcinoma model but had no effect in a preventive setting (91).

### Redundancy in Angiogenic Signaling Pathways

Initially, the primary focus in angiogenesis blockade was to target VEGF, which is the best known angio-stimulatory protein family responsible for EC activation and functional vessel formation and stabilization. Cancers that are highly dependent on the induction of angiogenesis by VEGF, were the best responders to anti-VEGF agents. These include CRC, RCC, and neuroendocrine tumors (139).

Cancers relying on angiogenic factors other than VEGF are less susceptible to anti-VEGF agents and include malignant melanoma, pancreatic cancer, breast cancer, and prostate cancer (98). The presence of several anti-VEGF resistant cancers suggests alternative angiogenic pathways. These involve Ang-1, EGF, FGF, granulocyte colony-stimulating factor (G-CSF), hepatocyte growth factor (HGF), insulin-like growth factor, PDGF, PGF, stromal cell-derived factor-1 (SDF-1), and TGF (140). Except for P1GF, which binds VEGF receptors, most angiogenic factors signal through specific transmembrane receptors, which are expressed on ECs (141). This variety of growth factors culminates in a plethora of pathways that tumor cells can exploit to induce angiogenesis.

Results from preclinical models and clinical trials suggest that inhibition of a specific growth factor can induce the expression of others (140, 141). In a study by Willett et al. in which rectal cancer patients were treated with BVZ, significantly increased plasma levels of PlGF were noted 12 days following the start of treatment (142). In a phase II study by Kopetz et al. in which metastatic CRC patients were treated with a combination of FOLFIRI and BVZ, the levels of several angiogenic factors including PlGF and HGF were found to increase before disease progression (54). Similarly, the levels of FGF2 and PlGF increased in GBM patients following treatment with cediranib, a pan-VEGF receptor tyrosine kinase inhibitor (71, 143). Similarly, treatment of transgenic mouse models of pancreatic tumors with an anti-VEGFR2 antibody for a prolonged period of time, associated with an increase in the expression of the pro-angiogenic growth factors, Ang-1, Ephrin-A1, Ephrin-A2, and FGF1, FGF2a, resulting in transient tumor growth delay and modest survival benefit (98, 144).

Redundancy in angiogenic signaling and potential in malignant tissues is nowadays more studied. In addition, the therapeutic effect of targeting a single angiogenic growth factor or its receptor became limited due to intrinsic resistance. This resistance arose either from redundancy in activated pathways or alternative growth factor signaling pathways. Thus, targeting multiple growth factors simultaneously or sequentially would be a successful approach to overcome such resistance. In the following subsection, we discuss potential angiogenic factors that might play a role in the escape from anti-VEGF treatment. We also shed light on results of studies evaluating the effects of targeting one or more of these factors on overcoming resistance to anti-VEGF therapies.

#### Angiopoietin

##### Role of angiopoietin in the escape from anti-vascular endothelial growth factor treatment

Ang-Tie signaling system is a vascular-specific RTK pathway that regulates vascular permeability and blood vessel development and remodeling through Ang-1 and Ang-2. Ang-1 binds to the Tie2 receptor on the M2 subpopulation of monocytes, HSCs, and ECs of blood and lymphatic vessels. This activates the Ang-Tie pathway and results in the maturation or stabilization of blood vessels (145). In contrast, Ang-2 blocks this pathway resulting in the remodeling or initiation of vascular sprouts following exposure to VEGF (146). Upregulation of Ang-2 expression was described in many types of cancers and presumable contributes to resistance against anti-VEGF therapy (147–151). For example, in CRC patients, elevated serum Ang-2 levels were associated with a poor response to BVZ treatment (152).

##### Targeting angiopoietin to overcome resistance to anti-vascular endothelial growth factor treatment

Blockade of both, VEGF and Ang2, in preclinical studies suppressed revascularization and tumor progression of cancers resistant to anti-VEGF therapy (92–95). However, results of ongoing clinical trials evaluating the efficacy of the humanized bi-specific monoclonal antibody against VEGF-A and Ang-2, vanucizumab, are still pending (153, 154).

#### Bombina Variegate Peptide 8 (Bv8)

##### Role of bombina variegate peptide 8 in the escape from anti-vascular endothelial growth factor treatment

Tumor-infiltrating T helper type 17 (Th17) cells produce interleukin-17 (IL-17), initiating a paracrine network to confer resistance to anti-VEGF therapy (38). IL-17 induces G-CSF secretion by tumor cells through nuclear factor κB (NF-κB) and ERK signaling (155). The increase in G-CSF induces the expression of Bv8, also known as prokineticin-2, in the bone marrow. Bv8 is a pro-angiogenic growth factor that was initially purified from the skin secretion of a yellow-bellied toad. It binds to the G-protein coupled prokineticin receptor (PROKR) and activates the downstream MAPK/ERK pathway (156, 157). As such, Bv8 promotes differentiation of myeloid-derived **(**suppressor) stem (remove word stem) cells (MDSCs) and induces their mobilization to the peripheral blood and infiltration into the tumor microenvironment. This culminates in the promotion of angiogenesis and results in the escape from anti-VEGF therapy (158–161).

##### Targeting bombina variegate peptide 8 to overcome resistance to anti- vascular endothelial growth factor treatment

Treatment with the Bv8 antagonist, PKRA7, suppressed tumor formation *in vivo* by inhibiting angiogenesis in GBM and infiltration of MDSCs in pancreatic cancer (96). Neutralization of Bv8 and upstream G-CSF using monoclonal antibodies also resulted in tumor suppression (162). Results of ongoing clinical trials evaluating combination regimens using Bv8 inhibitors with or without other anti-angiogenic reagents are still pending.

#### Fibroblast Growth Factor (FGF)

##### Role of fibroblast growth factor in the escape from anti-vascular endothelial growth factor treatment

The FGF family consists of 22 members. Four of these are intracellular cofactors of voltage-gated sodium channels, while the remaining 18 members are secretory proteins that bind to RTK–FGF receptors (FGFRs) (163). FGFR is expressed on tumor cells and several types of stromal cells, including cancer-associated fibroblasts (CAFs), ECs, and tumor-infiltrating myeloid cells (164).

Binding of FGF to RTK–FGFR activates the downstream pathways such as MAPK/ERK, PI3K/Akt, STAT, and diacylglycerol (DAG)/protein kinase C (PKC) (165–168). One of the roles of this signaling pathway is cancer development and progression through the amelioration of angiogenesis (164, 169). Indeed, upregulation of FGF2 expression correlated with resistance to anti-VEGF agents in several tumors resistant, especially those exposed to hypoxic environments (54, 71, 98, 170).

##### Targeting fibroblast growth factor to overcome resistance to anti- vascular endothelial growth factor treatment

Simultaneous blockade of VEGF and FGF signaling pathways was very beneficial in many preclinical models of cancer (98, 171–173). Combining the FGFR inhibitor, PD173074, with BVZ in xenografted mouse models with head and neck squamous cell carcinoma (HNSCC) completely abolished tumor growth (97). FGF blockade using the soluble FGF receptor, FGF-trap, was combined with an VEGFR2 inhibitor, and yielded comparable results in late stage pancreatic islet tumors (98). Unfortunately, in the clinical setting, patients with recurrence following anti-VEGF therapy did not benefit from the dual blockade of VEGFR and FGFR by dovitinib or nintedanib (99, 100).

#### Platelet-Derived Growth Factor

##### Role of platelet-derived growth factor in the escape from anti-vascular endothelial growth factor treatment

The PDGF family consists of four homodimers and one heterodimer. Binding of the PDGF dimers to tyrosine kinase PDGF receptor (PDGFR) results in the activation of downstream signal transduction pathways, such as PI3K and PLCγ (174). This plays an important role in mesenchymal cell growth and motility during embryonic development and tissue repair (175). When PDGF signaling is over-active in the tumor microenvironment, angiogenesis and tumor growth are promoted (176). Upregulation of PDGF-C expression was observed *in vivo* in CAFs infiltrating into tumors resistant to anti-VEGF therapy (101).

##### Targeting platelet-derived growth factor to overcome resistance to anti- vascular endothelial growth factor treatment

Sunitinib has many targets, including VEGFR and PDGFR. Following its FDA approval in 2006 for the treatment of metastatic RCC, it was assumed that combining PDGF and VEGF blockades might offer an additional therapeutic benefit (101). Several studies were initiated to evaluate the safety and efficacy of this combination (177). Unfortunately, combining BVZ with imatinib, which inhibits PDGF-R in addition to other tyrosine kinases such as Abl and Kit, was toxic and not effective treatment against RCC (102–104).

#### Transforming Growth Factor-β

##### Role of transforming growth factor-β in the escape from anti-vascular endothelial growth factor treatment

The TGF-β/Activin and bone morphogenetic protein (BMP) are the two main branches of the TGF-β superfamily. When TGF-β binds its type II receptors, it activates type I receptors and results in the phosphorylation of the receptor-regulated Smads (R-Smads) corresponding to each branch. R-Smads then complex with the common partner Smad4 (Co-Smad4) and work as transcription factors (178).

TGF-β signaling regulates cellular growth, differentiation, and apoptosis (179). Although signaling has tumor suppressive effects during the early stage, it switches toward malignant conversion and tumor progression at later stages (180, 181). It activates the production of extracellular matrix (ECM) by fibroblasts and stimulates tube formation by ECs, thus inducing angiogenesis (182–184).

Tumor tissues express higher levels of TGF-β and these levels can be correlated with patient survival (185–187). Upregulation of TGF-β expression was also observed in glioma models resistant to anti-VEGF therapy (188). This suggests a role of TGF-β in the acquired resistance to anti-angiogenic therapy.

##### Targeting transforming growth factor-β to overcome resistance to anti- vascular endothelial growth factor treatment

Several preclinical studies revealed the anti-angiogenic benefits when inhibiting TGFβ in CRC, HCC, and GBM xenografts (189–191). This offers the rationale to combine TGFβ inhibitors with anti-VEGF agents (192). In that sense, combining galunisertib, a small molecule inhibitor of TGFβRI, with sorafenib and ramucirumab in HCC is currently under evaluation (189, 193). Similarly, the combination of an anti-TGFβ monoclonal antibody, PF-03446962, with regorafenib in CRC is also under investigation (194).

#### Matrix Metalloproteinases

##### Role of matrix metalloproteinases in the escape from anti-vascular endothelial growth factor treatment

MMPs play an important role in angiogenesis and in different stages of cancer (195, 196). They are divided into six categories (Table 4) (197). MMP can promote or inhibit angiogenesis. For instance, the secreted MMP-9 plays an important role in the angiogenic switch process and in releasing VEGF from the ECM (1, 198). The membrane type MMP-1 induces degradation and remodeling of matrix during vascular injury and is responsible for invasion and migration of ECs and formation of capillaries (199–201). On the other hand, MMPs such as MMP-3, 7, 12, 13, and 20, inhibit angiogenesis through endostatin and angiostatin production. Endostatin that blocks the activation of pro-MMP-9 and inhibits capillary formation of Deryugina and Quigley (202).

**Table 4:** Categories of Matrix Metalloproteinase-1 and their corresponding members.

**Categories**	**Member(s)**
Collagenases	Matrix Metalloproteinase-1
	Matrix Metalloproteinase-8
	Matrix Metalloproteinase-13
Gelatinases	Gelatinase-A (Matrix Metalloproteinase-2)
	Gelatinase-B (Matrix Metalloproteinase-9)
Stromelysins	Matrix Metalloproteinase-3
	Matrix Metalloproteinase-10
	Matrix Metalloproteinase-11
Matrilysins	Matrix Metalloproteinase-7
	Matrix Metalloproteinase-26
Membrane-type matrix metalloproteinases	Matrix Metalloproteinase-14
	Matrix Metalloproteinase-15
	Matrix Metalloproteinase-16
	Matrix Metalloproteinase-17
	Matrix Metalloproteinase-24
Non-classified matrix metalloproteinases	

##### Targeting matrix metalloproteinases to overcome resistance to anti- vascular endothelial growth factor treatment

Targeting MMPs released by bone marrow derived cells (BMDCs) prevents the release of sequestered growth factors in the ECM, and can help overcoming resistance to anti-angiogenic therapy (203). Despite the fact that doing so has proven some clinical efficacy in patients with advanced and refractory solid tumors in a phase I clinical trial (105), most MMP inhibitors failed to offer any clinical benefit (204). Few agents are still being developed and evaluated. Results from an ongoing phase II clinical trial evaluating one MMP inhibitor in patients with Kaposi's sarcoma are still pending (205).

### Recruitment of Bone Marrow-Derived Cells

Long-term administration of AIs up-regulates HIF-1α and induces hypoxia in the tumor microenvironment by over-pruning blood vessels (206). Hypoxic conditions due to anti-angiogenic therapy result in the expansion and recruitment of myeloid cells and CAFs into the tumor environment. The presence of these BMDCs in the tumor microenvironment leads to a weakened antitumor response and an immunosuppressive tumor microenvironment (207). This promotes angiogenesis, tumor growth, EMT transition, and metastasis (208, 209). As a result, it has become evident that myeloid cells and CAFs play a major role in the induction of resistance to anti-angiogenic drugs.

#### Myeloid Cells

##### Recruitment of myeloid cells

Myeloid derived suppressor cells (MDSCs), also known as Gr1+ CD11b+ myeloid cells, consist of neutrophils, macrophages, and dendritic cells (DCs). An excessive production of MDSCs was described in cancer patients and tumor-bearing mice (210–213). This was linked to the immunosuppressive and tumor promoting capacities (214, 215). In a study by Shojaei et al., resistant tumors to anti-VEGF treatment had increased mobilization and infiltration of MDSCs into their microenvironments as compared with treatment-sensitive tumors (216).

Neutrophils are considered predictive biomarkers for patients treated with BVZ (217–222). Increased recruitment of neutrophils during anti-VEGF therapy promotes tumor progression and treatment resistance (216). This is mediated by the expression of the calcium-binding protein that regulates cell growth, survival, and motility, S100A4. As such, blocking granulocytes and S100A4 may be beneficial in diminishing anti-angiogenic therapy resistance (223).

Monocytes and macrophages are possibly implicated in resistance to anti-angiogenic therapy as well. Recruitment of these cells to the tumor microenvironment is mediated by different cytokines, including VEGF, chemokine C-C motif ligand 2 (CCL2), and macrophage colony stimulating factor (MCSF) (224, 225). Tumor associated macrophages actively participate in vascular sprouting by functioning as bridging cells between two different tip cells (226–228). They also secrete MMPs, promotingangiogenesis (198, 226, 229, 230). In addition, they can release pro-angiogenic growth factors including TGF-b, VEGF, EGF, and the chemokines, CCL2 and CXCL8 (226, 227, 231–233).

In different murine tumor models, anti-VEGF therapy reduced macrophage infiltration (217, 234–236). However, this was not the case with the tyrosine kinase with immunoglobulin-like and EGF-like domains 2 (TIE2)-expressing macrophages that constitute a specific subset of macrophages. These are usually recruited by HIF1a and tumor-secreted chemokines such as ANG2 in the setting of anti-angiogenic therapy (237–240). They tend to associate with tumor vessels and release proangiogenic growth factors including VEGF (237, 241). As such, macrophages contribute to the resistance against anti-angiogenic therapy. Preclinical studies on models of mammary carcinoma and insulinoma evaluated the effect of inhibiting ANG2 on TIE2-expressing macrophage infiltration and angiogenesis. Although this approach did not block the recruitment of these macrophages, it hindered the upregulation of their TIE2 receptor. This reduced the production of pro-angiogenic growth factors and the association of TIE2 macrophages with blood vessels (242–244). As a result, MDSCs represent promising targets for therapy. Since G-CSF expression stimulated by tumor infiltrating T helper type 17 cells results in MDSC recruitment into the tumor microenvironment, inhibition of Th17 cell function might sensitize tumors to anti-VEGF therapies (155, 207).

##### Targeting myeloid cells to overcome resistance to anti- vascular endothelial growth factor treatment

Since SDF1 is the major BMDC recruiting factor, targeting its signaling pathway could potentially decrease BMDC infiltration and overcome resistance to anti-angiogenic therapy. In a transgenic mouse model of breast cancer, treatment with an SDF1 neutralizing antibody inhibited MDSC infiltration and angiogenesis (106). Since Bv8 leads to the recruitment of MDSCs into the tumor tissue after VEGF blockade, its inhibition can possibly improve the effect of anti-angiogenic therapy. A recent study showed that the combination of gemcitabine and an anti-Bv8 monoclonal antibody treatment in mice with adenocarcinoma inhibited tumor regrowth, angiogenesis, and metastasis (107). In addition, anti-Bv8 antibodies blocked MDSC recruitment and tumor angiogenesis in an RIP1-Tag2 insulinoma model of pancreatic cancer (245).

Blocking the recruitment of monocytes and macrophages can be another therapeutic opportunity to overcome resistance to anti-angiogenic therapy. In a phase I clinical trial, patients with solid tumors were treated with the human anti-CCL2 monoclonal antibody, carlumab, which targets the monocyte chemotactic protein-1 (MCP1). In addition to causing a drop in free CCL2 levels and a reduction in the level of tumor-infiltrating macrophages, this therapy resulted in a temporary antitumor activity (108). Treatment of RIP1-Tag2 pancreatic neuroendocrine tumors with combined ANG2 and VEGFR2 blockers decreased infiltration of TIE2 expressing monocytes and suppressed revascularization and tumor progression (92). Since macrophages express colony stimulating factor-1 receptor, its targeting is currently being evaluated by several phase I clinical trials (NCT01346358; NCT01004861; NCT01596751). This is supported by results from earlier studies showing a reduced macrophage infiltration into tumor tissue and clinical objective responses following treatment of diffuse-type giant cell tumor patients with the anti-colony-stimulating factor-1 receptor antibody, RG7155 (246).

Macrophage Migration Inhibitory Factor (MIF) suppresses the anti-inflammatory activity of macrophages. TAMs, mainly M2-polarized macrophages, stimulate angiogenesis thus promoting tumor cell migration and progression (247). VEGF increases MIF production in a VEGFR-dependent manner. Compared to tissue specimens of BVZ-sensitive GBM patients, BVZ-resistant ones had a decreased MIF expression and an increased TAM infiltration (248). As such, blocking the VEGF pathway using BVZ can deplete MIF expression. This explains the enhanced recruitment of TAM and M2 in BVZ-resistant GBM tumors. Data is lacking when it comes to evaluating the application of this target in the clinical setting.

#### Endothelial Progenitor Cells

##### Recruitment of endothelial progenitor cells

Anti-angiogenic therapy causes hypoxia which results in the activation of HIF1a in tumor cells (249). This causes tumor cells to secrete SDF1 and VEGF,main chemotactic factors for EPCs (209, 215, 250, 251). Upon stimulation of the C-X-C chemokine receptor-7 (CXCR7) by SDF1, EPCs secrete pro-angiogenic cytokines and promote angiogenesis (252, 253). For instance, in multiple myeloma, this occurs through regulating the trafficking of angiogenic mononuclear cells into areas of tumor growth (254). EPCs can also promote angiogenesis by differentiating into ECs and subsequently incorporating into newly forming blood vessels.

### Recruitment of Local Stromal Cells

#### Pericytes

##### Recruitment of pericytes

Pericytes, also known as Rouget cells, are cells that interact with ECs. They regulate endothelial proliferation and differentiation and modulate vessel diameter and permeability, thus stabilizing the newly formed endothelial tubes (255, 256). In a study by Abramsson et al., paracrine co-signaling *via* PDGF-B and PDGFR-b played a major role in pericyte recruitment to ECs (257).

Several studies revealed enhanced pericyte recruitment to and coverage of the microvasculature in the tumor after treatment with AIs. Reduction in tumor vascularity following anti-VEGF therapy is accompanied by a tightly pericyte covered vessels (258). For instance, after treatment with sunitinib and the chemotherapy drug, temozolomide, a preclinical malignant glioma model revealed an increased number of vessels covered with pericytes (259). In addition, esophageal and ovarian cancer xenografts showed increased pericyte coverage around vessels following treatment with BVZ (260).

Tumor vessels that are heavily covered by pericytes have a reduced sensitivity for anti-angiogenic therapies (261) As such, the increase in pericyte infiltration was suggested to be a mechanism of resistance to anti-VEGF and anti-VEGFR therapies. By suppressing EC proliferation and by providing survival signals that contribute to the maintenance of ECs, pericytes mediate vascular maturation and stability hence allowing tumor cells to proliferate during the course of an anti-angiogenic therapy (262–264). As a result of protecting ECs from anti-angiogenic agents, pericytes were implicated in clinical resistance to VEGFR inhibitors (249).

While there is a broad consensus on the fact that pericyte-covered vessels are less sensitive to AI, several recent studies have highlighted that tumor vessels typically lack pericyte coverage due to their immaturity and rapid growth phase while normal quiescent vessels are well covered (265–267). This could identify a selective therapeutic window to target abnormal tumor blood vessels, rather than suggesting to target pericyte coverage.

In keeping with that, accumulating evidence supports the idea that—in addition to pruning non-covered vessels- cancer therapies should aim at promoting the establishment of a normal vasculature in tumors in order to favor wide distribution of standard chemotherapeutics and innovative drugs into the tumor mass and improve radiotherapy efficacy. This process is known as “vascular normalization” that many adopt as the future of anti-angiogenic therapy. By therapeutically improving, rather than reducing, the stability and function of tumor blood vessels, these may be exploited for delivery of therapeutics including endogenous anti-cancer immune cells. This would also improve perfusion, reduce hypoxia, and thereby reduce metastasis. Tumor vessel normalization for cancer therapy has been achieved by the application of molecules directly targeting endothelial cells, such as semaphorins (268, 269).

Although ANG1 is a growth factor that provides ECs with survival signals, its introduction in CRC tumor cells displays an anti-angiogenic therapy in one study (270). Although this approach was accompanied by a major increase in tumor microvessel pericyte coverage, it resulted in smaller tumors with less vasculature, suggesting a decreased sensitivity for angiogenesis (270). In a more recent study, tumor-bearing mice were treated with antibodies against ANG2A, and a similar observation was noted (261). Combining the chemotherapeutic agent, topotecan, with pazopanib significantly inhibited tumor growth, despite an increase in the number of vessels that were infiltrated by pericytes (271). Similar results were observed in a preclinical malignant glioma model following treatment with the combination of temozolomide and sunitinib (272).

##### Targeting pericytes to overcome resistance to anti- vascular endothelial growth factor treatment

Targeting blood vessel maturation by inhibiting pericyte coverage of the tumor vasculature was suggested as a promising strategy, to break the resistance to anti-angiogenic therapies and improve their efficacy. ECs secrete PDGF-B that mediates migration and proliferation of pericytes expressing PDGFR-b (273). Since SDF1, and the heparin-binding EGF-like growth factor also play a major role in pericyte behavior (274), blocking the PDGF pathway alone might not be sufficient to prevent pericyte coverage of vasculature.

Although several studies showed that targeting pericytes and ECs leads to impaired tumor growth and improved efficacy to anti-angiogenic agents, data negating the potentiation of treatment outcome with dual blockade exists (275). For instance, in a study by Nisancioglu et al., treatment of lung cancer in pericyte-deficient PDGF-B (ret/ret) mice with the anti–VEGFA antibody, G6-31, did not have any additional anti-tumor benefit (276).

Other pathways like sphingosine-1-phosphate (S1P)/edg-1, TGF-b1/Alk5, or MMPs should be considered while trying to overcome resistance associated with pericyte coverage (277). As a result, anti-pericyte agents should always be combined with other therapies, including chemotherapeutic agents. For instance, in a preclinical study by Pietras et al., transgenic mouse models of cancer were treated with a combination of the two anti-PDGFR agents, imatinib and SU11248, cyclophosphamide, and/or an anti-VEGFR agent (109). Compared to monotherapies, combination therapies significantly improved anti-tumor responses. Of note, the combination of all three approaches resulted in complete responses. Also, treatment of neuroblastoma mouse xenograft models with a combination of metronomic topotecan and pazopanib resulted in a sustained anti-angiogenic effect. but induced resistance mediated by elevated glycolysis (109).

#### Cancer-Associated Fibroblasts

##### Recruitment of cancer-associated fibroblasts

CAFs are activated by growth factors released from tumor and inflammatory cells, including TGFb, PDGF, and FGF (169, 278, 279). CAFs also secrete several pro-angiogenic growth factors, including EGF, HGF, and FGF. For instance, VEGF-producing CAFs maintain tumor angiogenesis in VEGF-deficient tumor cells (280).

When CAFs were isolated from a mixture of EL4 tumors resistant to anti-VEGF agents and TIB6 tumors sensitive to anti-VEGF agents, they were able to promote tumor cell proliferation and growth even when VEGF was blocked. When CAFs were isolated from TIB6 tumors sensitive to anti-VEGF agents, no tumor growth was observed (215). This supports the role of CAFs in the acquired resistance to anti-angiogenic therapy. Further analysis revealed an upregulation in the expression of pro-angiogenic genes in CAFs derived from therapy-resistant tumors, and these included PDGF-C and Ang-like protein 2. As a result, it is assumed that a PDGF-C neutralizing antibody could be used in the treatment of tumors refractory to anti-VEGF agents (215).

CAFs can promote tumor growth and angiogenesis through the release of certain growth factors and proteases. For instance, CAFs secrete the chemokine SDF1 which directly stimulates tumor cells and recruits EPCs and other BMDCs into the tumor tissue (250, 251). They also produce proteases, including MMPs that stimulate the release of matrix-bound pro-angiogenic growth factors, thus promoting angiogenesis and resistance to anti-angiogenic agents (281–283).

##### Targeting cancer-associated fibroblasts to overcome resistance to anti- vascular endothelial growth factor treatment

Targeting CAFs might play a role in overcoming resistance to anti-angiogenic therapy. Treatment of nude mice human HCC xenografts with the anti-FGF2 monoclonal antibody, GAL-F2, inhibited tumor growth and angiogenesis by blocking the effect of the proangiogenic FGF in CAFs. Also, the addition of an anti-VEGF antibody or the tyrosine kinase inhibitor, sorafenib, led to an additive treatment effect (110). Similarly, treatment of patients with recurrent and persistent endometrial cancer with the dual VEGFR/FGFR inhibitor, brivanib, extended their progression-free survival (PFS) by blocking the effect of the proangiogenic VEGF in CAFs. (111). Neutralization of PDGF-C suppressed CAF-mediated tumor progression.

### Adoption of Different Neovascularization Modalities

Besides acquiring resistance to angiogenesis inhibition through growth factor redundancy and recruitment of different cells, tumor cells may also escape the effect of AIs by adopting different neovascularization modalities (284–286). These include vascular co-option and vasculogenic mimicry.

#### Vessel Co-option

##### Role of vessel co-option in the escape from anti-vascular endothelial growth factor treatment

Vessel co-option refers to the process by which cancer cells incorporate into and grow along pre-existing vessels rather than inducing new vasculature (287). This strategy provides oxygen and nutrients for efficient tumor outgrowth. It was first described in brain tumors arising from well-vascularized brain parenchyma (288). For instance, vessel co-option was also observed in gliomas and other cancer types including lung cancers (289). It was shown to sustain the growth of cerebral metastases from melanomas, liver metastases from breast cancers and NSCLCs, and lung metastases from different primaries (290, 291). Interestingly, vessel co-option is independent of the classic angiogenic switch and doesn't require any angiogenic growth factors. As such, vessel co-opting tumors are usually not sensitive to anti-angiogenic agents. For example, patients with CRC and liver metastases demonstrated a poor response to BVZ therapy due to vessel co-option.

An interesting question is whether this process represents an intrinsic resistance mechanism to anti-angiogenic therapies or whether it occurs in response to treatment. According to results from several studies, an increase in vessel co-option tends to follow, rather than precede, the inhibition of angiogenesis (292). For instance, the use of an anti-VEGF antibody in GBM patients resulted in an increase in vessel co-option (293, 294). Similarly, the growth cerebral melanoma metastasis was sustained by vessel co-option following treatment with the anti-angiogenic agent, ZD6474 (290). Nevertheless, more data is needed to check whether this applies to different tumor types and to evaluate its impact in the clinical setting.

#### Vasculogenic Mimicry

##### Role of vasculogenic mimicry in the escape from anti-vascular endothelial growth factor treatment

Vasculogenic mimicry refers to the process in which vascular-like structures are formed by tumor cells, after they trans-differentiate and gain features of ECs such as the expression of the endothelial markers, VE-cadherin, TIE1, and ephrin A2 (295, 296). Since no new blood vessels are formed, this phenomenon is different from vasculogenesis and angiogenesis. Nevertheless, the fact that blood can still be transported through the vascular-like networks and tumors can still be well-oxygenated, vasculogenic mimicry strongly associated with poor patient survival. This process was described in different tumor types, including gliomas, malignant melanomas, sarcomas, and breast cancers (284, 297–300).

Since tumor cells trans-differentiate into endothelial-like cells as part of vasculogenic mimicry, it might be assumed that the process can be inhibited by anti-angiogenic agents. However, tumor cells that make use of this phenomenon were not found to develop sensitivity to anti-angiogenic therapies in early studies (301). Instead, they were shown to upregulate this process following treatment with BVZ or induction of hypoxia by several preclinical studies (302, 302, 303). As such, vasculogenic mimicry might serve as an escape mechanism from anti-angiogenic therapies. The idea of combining AIs with chemotherapeutic agents has been suggested but more data is needed to evaluate its impact in the clinical setting.

##### Targeting vasculogenic mimicry to overcome resistance to anti- vascular endothelial growth factor treatment

Following the emergence of vasculogenic mimicry as an alternative vascular-like network in tumors, researchers have realized the importance of combining angiogenesis inhibition with an anti-tumor cell strategy. This is particularly challenging because the transition of tumor cells into a more stem cell–like phenotype is linked to reduced responsiveness to chemotherapy and radiotherapy.

In an attempt to better understand the regulators of vasculogenic mimicry, several studies tried to recognize the molecular players of this process. Direct targeting of these molecules, including VEGF, is thought to serve as a promising therapeutic approach (302, 303). Other regulators of mimicry were also involved in the plasticity and stem cell-like phenotype of tumor cells. An example is the overexpression of the marker of brain development, NODAL (304–307). In addition, the overexpression of CD44 on vasculogenic tumor cells led to the initiation of the ongoing clinical study (NCT01358903). This trial evaluates the effect of an anti-CD44 agent on the process of vasculogenic mimicry during the treatment of solid tumors.

## Conclusion and Future Outlook

The concept of targeting tumor angiogenesis is an important advancement in cancer therapy and has resulted in the development of therapeutic agents such as BVZ, sunitinib, and sorafenib. Benefits of using anti-angiogenesis therapies seem to be limited due to several reasons.

Despite the resulting stabilization of disease and increased PFS, treatment with anti-angiogenic agents may give rise to more resistant tumors with higher patient relapse rates. This lack of clinical benefit could be associated with preexisting resistance or with rapid adaptation to anti-angiogeneic agents. It is clear that multiple mechanisms of resistance against AIs exist, including upregulation of alternative angiogenic factors by tumor cells, involvement of stromal cells, and co-option/mimicry. The fact that the process of angiogenesis is complicated and involves a network of mechanisms suggests that the tumor microenvironment could mediate resistance to AIs (308). In addition, the vascular regression that is caused by AIs could elevate intra-tumoral hypoxia, which in turn, ameliorates resistance to radiotherapy, chemotherapy, and AIs. Also, the regression in tumor vasculature and the reduction in blood flow that result from AIs would impede the delivery of chemotherapeutic agents into tumors. All these complications of AI use would allow for tumor metastasis and would hence serve as practical limitations to drug development (309).

With the progress in several scientific and medical fields and with the growing surge in knowledge about angiogenesis and its resistance mechanisms, new pharmacological strategies ought to be developed in the near future. For instance, new ways of targeting tumor vessels should be designed. This could be made possible by developing novel therapeutics that can either optimize the function of tumor vessels to allow adequate tumor response to cancer therapy or directly target tumor vessels (310).

In addition, in the light of the wide gap between our improving knowledge in the mechanobiology of MSCs and our satisfactory understanding of their clinical implications, novel approaches should be suggested to fill the gap. This could be made possible by engineering MSCs to selectively deliver anti-angiogenic molecules (309).

In addition, the use of combination strategies as a means to target multiple pathways involved in angiogenesis has been suggested to be a promising approach in overcoming resistance to AIs. To date, these either include a combination of multiple anti-angiogenic agents or a combination of anti-angiogenic drugs and other treatment regimens.

This process of selecting the most effective combination regimen is challenging because it requires extensive profiling of angiogenesis signaling pathways and involves a careful patient selection. Not only do combination regimens require regular dose adjustments to enhance efficacy and reduce toxicity, but also they require intermittent monitoring of treatment efficacy through biomarkers. Although combinations of different anti-angiogenic agents might increase treatment benefit, the presence of many alternative pathways can still result in acquired resistance. They can also induce excessive hypoxia that leads to additional resistance. Hence, the initiation of clinical trials to evaluate the efficacy and safety of such new combination strategies seems to be of utmost importance. In addition, the development of genetically engineered animal models whose tumor microenvironment can mimic that of humans could be of so much help in the development of reliable treatment approaches. This, in addition to clinical trials, would enable scientists and clinicians to make use of precision medicine for coming up with effective combinations of AIs and other therapies that would hopefully prevent the early acquisition of resistance or even impede its occurrence (141).

It is likely that the future therapy will make use of genomic, transcriptomic, and proteomic techniques as part of diagnostic profiling. Different therapeutic combinations can then be personalized and matched to current stages of tumor progression. Since tumors have rapid genetic drifts and might rapidly develop resistance to treatment, diagnostic profiling would have to be repeated during the course of treatment (141).

Nanotechnology enables researchers to develop novel nano-therapeutics, but this requires more knowledge about metabolic behaviors of tumor cells and possible physiological barriers or material properties that would improve or impede the efficiency of nano-therapeutics, respectively (311). It can therefore be foreseen that the future of AI-based therapies is heavily dependent on the efforts of basic scientists who can provide a clearer image regarding the response of cancer cells to the agents and on the ability of clinicians to make use of this knowledge to benefit patients (312).

These issues highlight the major challenges for future research. We look forward to the results of ongoing and future clinical trials discussed in this review paper in hopes that outcomes can be improved for all patients with cancers that are resistant to angiogenesis.

## Author Contributions

All authors made substantial contributions to study conception and design. YH, MK, HE, IK, DM, ST, and AS have been involved in drafting the manuscript and revising it critically for important intellectual content. All authors have provided final approval of the version to be published.

### Conflict of Interest

DM reports honoraria, travel support and institutional research funding from Roche, Pfizer, Novartis, and Amgen. The remaining authors declare that the research was conducted in the absence of any commercial or financial relationships that could be construed as a potential conflict of interest.
